# CellWHISPER disentangles direct cell–cell communication from structural proximity

**DOI:** 10.64898/2026.01.07.697982

**Published:** 2026-10-22

**Authors:** Anurendra Kumar, Felix Rivera, Bhavay Aggarwal, Nicholas Zhang, Ahmet Coskun, Saurabh Sinha

## Abstract

Spatial transcriptomics makes it possible to infer direct, contact-dependent cellular communication, but existing tools are prone to high error rates due to spatial structure in cell types and gene expression. We present CellWHISPER, a framework for inferring contact-mediated communication, including gap junctions and ligand-receptor signaling, from spatial transcriptomics data. CellWHISPER controls errors with a statistical procedure that accounts for confounding effects of cell-type-specific expression and spatial organization. Closed-form significance scores enable analysis of large data. A latent variable model distills recurrent patterns into maps of preferences among cell types and signaling genes. We demonstrate CellWHISPER on two technologies (Stereo-seq, Xenium), two species and three tissues. In mouse brain it yields a comprehensive connexin-interaction map. Immunostaining confirmed CellWHISPER-predicted Connexin 43-mediated interfaces among astrocytes, microglia and endothelial cells, which existing approaches fail to prioritize. In human colon and skin, tissues with strong spatial gradients, CellWHISPER recovers communication mechanisms both along and independent of the gradient.

